# SEGCN: a subgraph encoding based graph convolutional network model for social bot detection

**DOI:** 10.1038/s41598-024-54809-z

**Published:** 2024-02-19

**Authors:** Feng Liu, Zhenyu Li, Chunfang Yang, Daofu Gong, Haoyu Lu, Fenlin Liu

**Affiliations:** 1 School of Cyber Science and Engineering, Zhengzhou University, Zhengzhou, 450002 China; 2 Henan Provincial Key Laboratory of Cyberspace Situational Awareness, Zhenzhou, 450001 China

**Keywords:** Computer science, Information technology

## Abstract

Message passing neural networks such as graph convolutional networks (GCN) can jointly consider various types of features for social bot detection. However, the expressive power of GCN is upper-bounded by the 1st-order Weisfeiler–Leman isomorphism test, which limits the detection performance for the social bots. In this paper, we propose a subgraph encoding based GCN model, SEGCN, with stronger expressive power for social bot detection. Each node representation of this model is computed as the encoding of a surrounding induced subgraph rather than encoding of immediate neighbors only. Extensive experimental results on two publicly available datasets, Twibot-20 and Twibot-22, showed that the proposed model improves the accuracy of the state-of-the-art social bot detection models by around 2.4%, 3.1%, respectively.

## Introduction

With the rapid development of Internet technology, cybersecurity incidents in cyberspace are frequent and have a great impact. Countries around the world regard network security situation awareness as the key to the strategic layout of cybersecurity. As an indispensable part of cyberspace, the security of online social networks has aroused widespread concern. Social bots pose a great challenge to online social network security. Social bots are social accounts that controlled by automated programs^1^. Bot manipulators use bots to perform various malicious activities in social networks (e.g., spreading rumors^1,2^, polarizing online discussions^3^, amplifying popularity^4,5^, etc.) seriously jeopardize the security of cyberspace and cause adverse effects on society. Social bots have been found in different domains, including politics^6^, health^7^, and business^8,9^.

As social networks become increasingly connected to people’s lives, we are vulnerable to potential manipulation by bots. For example, in Mumbai, social bot spread rumors on social media that the vaccines were a plot by the government to sterilize Muslim children, which led to that only 50% of those who were expected to be vaccinated actually got the vaccine^7^. During the 2016 U.S. election bots posted numerous smear tweets against their competitors, swaying their supporters^6^.

Social platforms and researchers proposed a series of social bot detection models to minimize the impact of malicious social bots, with early success. These detection methods can be grouped into two categories—account feature-based methods and graph structure-based methods.

Existing feature-based detection methods for social bot use many hand-crafted features from different categories of information, such as profiles, content, networks, properties and train machine learning models to separate the bots from benign users. However, many of the existing features are effectless when facing the manually aided created profile property and scheduled activities of social bot generated by complex stochastic algorithms. For instance, the rapid development of deep forgery techniques allows social bots to have identical profile information as normal accounts and automatically establish social relationships with other accounts, interspersing small amounts of malicious information with many neutral ones, which is very different from the traditionally considered bot behavior^10^. The study on Twitter bots^11,12^ indicates that current social bots can more delicately disguise themselves as normal accounts and work in concert to achieve certain specific purposes, such as spreading rumors, posting advertisements.

To address these challenges, several studies used the interactions of accounts in social networks to construct social graphs which are then divided into cohesive subgraphs using graph mining techniques. This type of approach usually considers only leveraging the links of social bots in online social networks but misses the automated cues embedded in the text, time, and profile information. Therefore, these methods are unable to detect social bots that have successfully established enough attack edges (links) with normal users^13^.

Inspired by graph neural network (GNN) models that utilize both structural and property features of nodes, some researchers used GCN, graph attention networks (GAT), and other Message passing neural networks (MPNN) models to integrate account property features and structural features for social bot detection^11,14^, with promising results. However, MPNN’s expressive power is upper-bounded by the 1st-order Weisfeiler–Leman (WL) isomorphism test^15^. The WL algorithm can be *k*-dimensional, which considers the *k*-tuple of the vertices when calculating the graph isomorphism problem. If only one vertex’s characteristics (such as labels, properties, etc.) are considered, then it is a 1-dimensional WL (1-WL) algorithm. The 1-WL algorithm results in a unique set of features on most graphs, which means that each node on the graph has a unique role positioning. Therefore, for most irregular graph structures, the features obtained using 1-WL algorithm can be used as the basis for determining whether the graph is isomorphic, that is 1st-order Weisfeiler–Leman test. Importantly, researchers found that such method cannot capture basic graph structure features such as cycles and triangles^16,17^. Yang et al.^18^ proposed that cycles or triangles are important features in social bot detection tasks.

To improve the expressive power of GCN, capture the basic structure in the graph, and improve the detection performance of the model, we design a subgraph encoding-based GCN model for social bot detection.

### Motivation

Bot operators are easily aware of the property features used by the bot detection model and they tend to evade detection by avoiding these features^10,18^. Social bot detection models using purely structural features are unable to detect social bots that have successfully established sufficient attack edges (links) with ordinary users^13^. The MPNN-based social bot detection model proposed by Feng et al.^11^ achieved good results in the social bot detection task, but it ignored the limitations of the expressive power of the MPNN.

In general, motivation can be summarized into the following two points:

- Basic graph structure features, such as rings and triangles, are important to detecting social bots. However, these features cannot be captured by directly using messaging neural networks in the entire social graph.
- Considering various types of features, rather than one type of features, can boost the social bot detection performance.

### Contributions

To address the above problem, we propose an end-to-end social bot detection model with combined account semantic features, property features and structural features. Specifically, first, we vectorize the semantic and property information of the account and concatenate them into the initial representation vector of the nodes. Then, a random walk is used to extract a fixed-length subgraph of each node, and the final representation of the node is obtained using subgraph encoding. Finally, Softmax is used to identify machine accounts and human accounts.

- A GCN-based social bot detection model is proposed. The model detects social bots using semantic features, property features, and structural features of accounts simultaneously.
- Improve the expressive power of the GCN by using subgraph encoding to capture differences in the basic structure (e.g., cycles or triangles, etc.) between accounts.
- We analyze the impact of different types of features on model performance. Extensive experimental results show that the proposed model achieves better performance compared to the state-of-the-art models.

## Related work

The earliest work on social bot detection dates back to 2010^19^, honeypot traps were designed to detect social bots. Over time, the development of social bots has shown two main trends: single-account feature-based social bot detection and groups-based one. This section introduces the characteristics of these two categories of methods.

### Single-account feature-based social bot detection

Early social bot detection methods were mainly based on feature engineering of account properties, using traditional classifiers for classification. The work from^20^ filters social bots by analyzing Twitter account profiles. Specifically, it designed 16-dimensional features, for instance, screen name length, active days, the number of posted tweets, by analyzing account properties, tweet content, historical activity, and friend lists. Afterwards, it feeds these features into a random forest classifier to distinguish bots from humans, which is one of the foundational work on social bot detection based on individual accounts. Many follow-up studies continue to mine more features from accounts to improve the accuracy of model detection^18,21,22^. Some researchers, considering that social accounts should not be classified only as bots and non-bots due to the hijacking of human accounts in social networks, studied the differences between humans, bots and cyborgs in terms of tweets (number of tweets, time of posts) and account properties (external URL ratio, account reputation, etc.)^23^. This work laid down the idea of designing different classifiers for different types of bots. Cresci et al.^24^ designed digital DNA, a string of characters that encodes the sequence of the accounts’ action, to train different classifiers to detect different bots.

However, over time, bot operators gradually learned about classical bot detection features and managed to evade detection. The traces of the continuous evolution of bots can be found from^10,11,18^. In response to this trend, researchers continue to exploit individual account features. Yang et al.^18^ mined 10 new features from the data, such as account clustering coefficients, two-way following ratio, and tweet similarity, to train classifiers against the evolution of bots. Beskow et al.^21^ extracted differentiated account profile features (degree centrality, K-betweenness centrality, mean eigen centrality, etc.) and tweet features (mean/max mentions, number of languages, etc.) from the collected data and used the random forest as the classifier. Subsequent researchers designed new features to combat the continuous evolution of bots and achieved good performance^25,26^. But it should be noted that the designed features are subject to the specific social platforms, which limits the generalization ability of these models.

To address the challenge of generalization ability and design generic social bot detection models, some researchers^27^ designed various classifiers for bots using different datasets and combined these classifiers into an ensemble; Botometer-v3^28^, a social bot detection system that incorporates 1700-dimensional features to improve generalization, boosted a series of research works on social bots detection^28–30^; Some scholars used natural language processing methods to extract semantic differences from account tweets to detect social bots. For example, the work from^31^ designed a long short-term memory network (LSTM) based model to extract content features and temporal features of tweets to distinguish bots and people. Pre-training models in natural language processing are also applied in social bot detection^11,32^.

The confrontation between bot detectors and operators is a never-ending race. The properties of a single account are easy to be forged and tampered. Dealing with this challenge, researchers work on group-based social bot detection methods.

### Group-based social bot detection

The group-based social bot detection method utilizes the structural differences between the social graphs generated by humans and bots. The relationships that are used to build the social graph are usually friend relationships^33^, following/follower^34,35^, retweet/retweeted^36^. The detection mechanism is to use the homogeneity of social networks, in another word, the neighbor nodes of the bot tend to be bots, and the neighbor nodes of the human tend to be humans^34,37–39^. A label-enhanced network integrates labels with social networks and uses the defined badness score based on the random walk of nodes to distinguish bot and human^39^. Wang et al.^38^ proposed paired Markov random field models to estimate the posterior probability of each user by loopy belief propagation and predict the user’s label based on the posterior probability. Moreover, they proposed a framework to unify random walk and loopy belief propagation in^37,40^ to address the limitation of the method^39^ that it cannot utilize the label of bot and human, meanwhile, avoiding the problems of the method^38^ that it is not scalable and does not guarantee convergence. The study from^41^ trained a local classifier to calculate the local trust scores of nodes and edges, and then the local trust scores used for prediction are propagated through the global network structure by a weighted random walk and loopy belief propagation mechanism.

These group-based social bot detection methods largely improve the generalization of the model and avoid manual feature engineering^42,43^. However, this type of method only utilizes the link information between accounts, and its detection performance is greatly reduced when enough attack links are established between accounts^13^.

With the rise of GCN^44^, it has been widely used in various occasions, such as link prediction, node classification, community division. Researchers introduce GCN to detect social bots because GCN can utilize the link information between accounts as well as lots of other information. Sun et al.^45^ designed a GCN with trust mechanism. First, the method starts a short random walk from a known real node, and its walk probability is the trust score of the node. Then, it uses these trust scores as edge weights, and uses graph convolution operations to aggregate features from local graph neighborhoods onto a weighted graph for classification. This work^14^ proposed a GCN-based spam bot detection model which utilizes both account property features and neighborhood features. Following this direction, researchers designed a social bot detection model using the semantic features and property features of the relational graph convolutional network (RGCN^46^). First, it vectorizes the property features and semantic features of the accounts and concatenates the two types of vectors together. Then, the spliced semantic vectors are fed into a neural network model for training to detect social bots. This method achieves state-of-the-art results on homogenous graph social bot detection.

Recently, RoSGAS^47^ designed an adaptive search GNN structure for social bot detection model, which gets rid of the a priori of people designing GNN structures and searches for appropriate GNN structures through reinforcement learning. RF-GNN^48^ utilized the idea of integrated learning to detect social bots by combining the Random Forest algorithm and GNN. They both directly aggregate information from the direct neighbors of the account, which may fails to capture the differences in the basic structures (rings or triangles, etc.).

## Proposed approach

**Figure 1:**
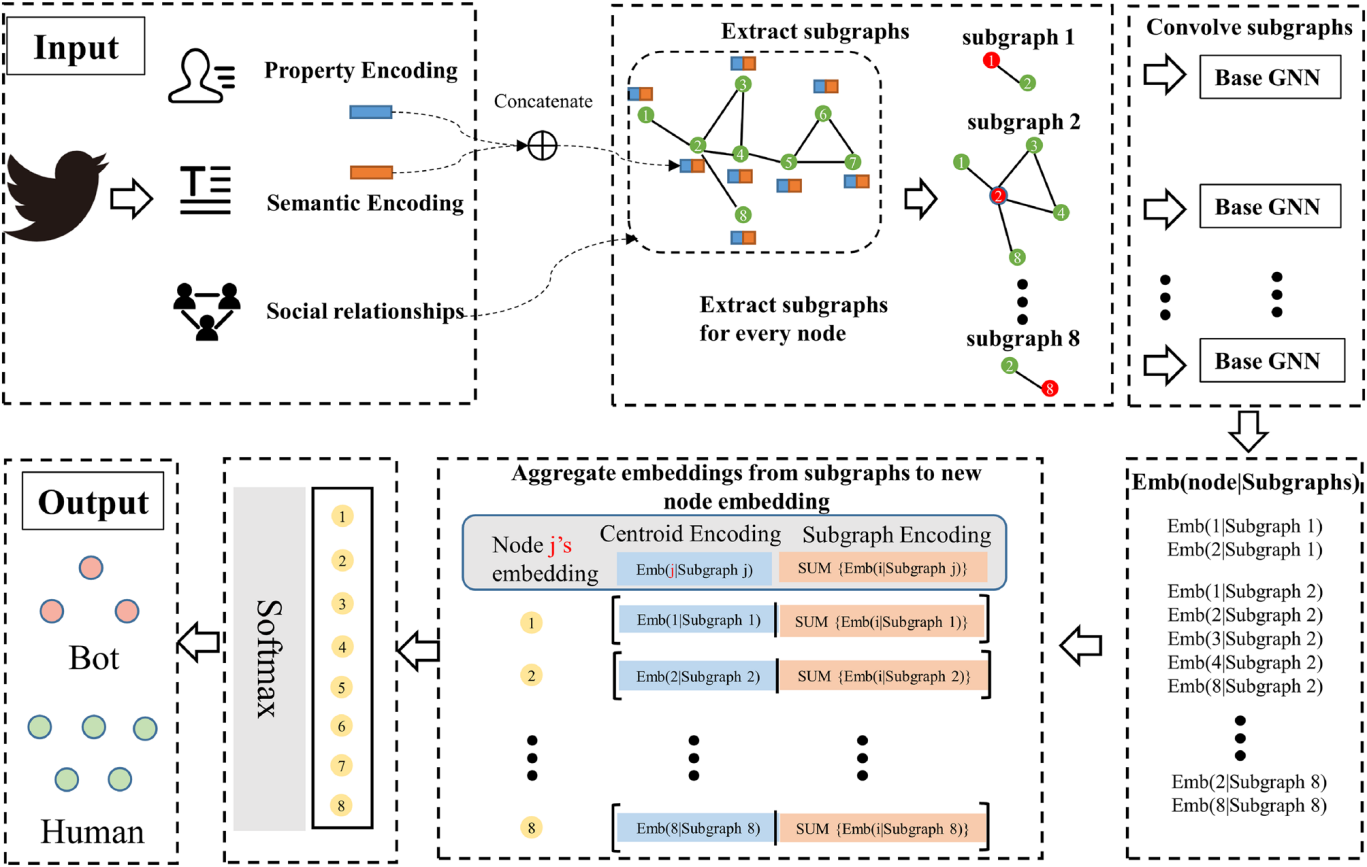
The framework of the proposed social bot detection model.

To address the above challenges, we design a subgraph encoding-based approach for social bot detection, dividing the social network into multiple subgraphs and coding each node in the subgraphs with a GCN, which significantly differs from the existing methods. Since a node may belong to multiple subgraphs, so there are multiple representation vectors of a node, which enhances the representation of the node. Compared to GCNs where the central node features originate from the aggregation of its immediate neighbors, subgraph encoding considers both immediate and non-immediate neighbors, making it capable of capturing basic structural information such as rings and triangles, and therefore, more suitable for social bot detection. This is the difference between our approach and existing social bot detection methods. The framework of our model is shown in Fig. 1 and the implementation details of the model are specifically described in the following subsections.

### Input

Social accounts contain abundant data information, and existing social bot detection methods identify bot accounts by mining the information contained in social accounts. This paper proposes to use account semantic features, property features, and structure features for learning account representation. The semantic features are extracted from the account’s descriptions and tweets. The account’s profile, such as account ID, screen name, profile image, is the source of the property features. The social graph whose edge represents the following and follower relationship between accounts is the input for extracting the structure features.

### Node representation

Learning the node (account) representation is a very important process for downstream tasks, and the node representation directly affects the model performance.

#### Semantic representation

Tweets can largely reflect the characteristics of the accounts and are widely used by the existing bot detection methods. We use the RoBERTa^49^ language model in Transformer.pipeline to encode account semantic information. The semantic feature vector $\varvec{N}_s^u$ for a given account *u* consists of two components: the account description semantic vector $\varvec{N}_d^u$, the tweet semantic vector $\varvec{N}_t^u$. The account description is a paragraph set by Twitter users to briefly introduce themselves.

First, we use RoBERTa to learn the representation vector $\varvec{N}_d^u$ of the *u*-th account description information (see as Eq. (1)),

1 $$\begin{aligned} \varvec{N}_d^u=\sigma (W_d\cdot \frac{1}{n}\sum _{i=1}^{n}RoBERTa(\{{d_i}\}_{i=1}^n)+b_d), \end{aligned}$$

where ${d_i}\in {\mathbb {R}}^{D_R\times 1}$ and $\{{d_i}\}_{i=1}^n$ is the *u*-th account description that consists of *n* words and *i* represents the index of the word in the description. $D_R$ is the embedding dimension which is predefined in RoBERTa. $W_d$ and $b_d$ are learnable parameters. $\varvec{N}_d^u\in {\mathbb {R}}^{D\times 1}$, *D* is the dimensionof the output vector of the MLP. $\sigma$ is the activation function. In this paper, Leaky-ReLU^50^ is used as the activation function.

The semantic vector of account tweets can be obtained in a similar method (see as Eq. 2)

2 $$\begin{aligned} \varvec{N}_t^u=\sigma (W_s\cdot \frac{1}{M_u}\frac{1}{m_u}\sum _{i=1}^{M_u}\sum _{j=1}^{m_u}RoBERTa(\{{w_j^i}\}_{j=1}^{m_u}+b_s), \end{aligned}$$

where $w_j^i\in {\mathbb {R}}^{D_R\times 1}$ and $\{{w_j^i}\}_{i=1}^{m_u}$ is the *i*-th word of the *j*-th tweet, and the tweet length is $m_u$. $W_s$ and $b_s$ are learnable parameters. $M_u$ is the number of tweets from the *u*-th account. $\varvec{N}_t^u\in {\mathbb {R}}^{D\times 1}$.

Combing the two parts obtained above, we can get the semantic feature vector of the *u*-th account, namely, $\varvec{N}_s^u=\left[ \varvec{N}_t^u,\varvec{N}_d^u\right] , \varvec{N}_s^u\in \ {\mathbb {R}}^{2D\times 1}$.

#### Property representation

Many early social bot detection studies were successful in distinguishing bot accounts from benign accounts based on the property features of the accounts^10,18^. In this paper, the properties of accounts are divided into statistical features (e.g., number of followers, likes, retweets) and category features (e.g., whether the account is authenticated, whether it uses default profile information, whether it displays location information). All the property features used for account representation are shown in Table 1. Concerning vectorization to the property features, we use Z-Score normalization for the numerical features and One-hot encoding for the category features. The processing details can be referred to^11^

**Table 1:** Property features used in our model.

Feature name	Description
Followers	The number of followers an account has
Followings	The number of accounts that the account follows
Favorites	The number of favorites or likes an account receives
Statuses	The number of statuses an account posts
Active days	The number of days from the account’s registration to current
Screen name length	The length of the account’s current screen name
Protected	Whether the account is currently protected
Contributors enabled	Whether contributors are enabled or not
Is translator	Whether there is a translator or not
Is translation enabled	Whether the translation is available or not?
Geo enabled	Whether the account is geo enabled or not
Background tile	Whether the account uses a background tile
Background image	Whether the account uses a background image
Extended profile	Whether the account has extended profile or not
Default profile	Whether the account uses a default profile
Default profile image	Whether the account uses a default profile image
Verified	Whether the account is verified or not

The property feature vector $N_p^u$ for the *u*-th account is combined as $\varvec{N}_p^u=[\varvec{N}_{P_n}^u,\varvec{N}_{P_c}^u],\varvec{N}_p^u\in {\mathbb {R}}^{2D\times 1}$.

Ultimately, the initial feature representation vector of account *u* can be expressed as $\varvec{N}_{init}^u=[{\varvec{N}_s^u},\varvec{N}_p^u], \varvec{N}_{init}^u\in \ {\mathbb {R}}^{4D\times 1}$.

#### Subgraph encoding

The core idea of subgraph coding is to obtain more expressive structural features of the whole graph by encoding the subgraphs extracted from the graph, which is similar to the idea of word segmentation in natural language processing. In this paper, GCN is used as encoding model for the subgraphs.

**Figure 2:**
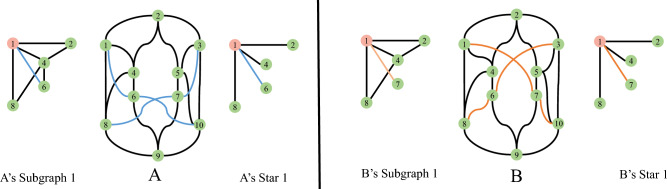
Illustration of the Two 4-regular graphs that cannot be distinguished by 1-WL. Colored edges are the difference between two graphs. There are differences in the first-order subgraph of some nodes in the graph.

Graph nodes contain rich structural and property information. In MPNNs, each node aggregates its neighbor features in a star pattern. Therefore, MPNNs cannot distinguish the non-isomorphic regular graphs with the same star structure^51^. However, two non-isomorphic graphs with the same star structure but their subgraphs may differ (see as Fig. 2). The star structure of node “1” in Fig. 2A,B are identical, but there are differences in their subgraph structures. Subgraphs retain basic structural features such as cycles or triangles.

##### Subgraph extraction

:n social networks, the *k*-hop egonet of a node as a subgraph may be too large^52^. Therefore, we use random walking to extract subgraphs that limit the subgraph size (see as Eq. (3)). In practice, We use the random walking rule in Node2vec [46].

3 $$\begin{aligned} G(N_{rw}(u)) ={Random\ walk}_{W_l}\ (u|u\in V), \end{aligned}$$

where $W_l$ is the random walking length. *u* is a subgraph root node. $N_{rw}(u)$ denotes the set of nodes visited by the random walker. $G(N_{rw}(u))$ denotes the subgraph whose root node is *u*.

##### Subgraph encoding

Subgraph encoding can improve the expressive power of GCN, and^51^ demonstrated both theoretically and experimentally that subgraph encoding surpasses 1-WL and 2-WL and can be no weaker than 3-WL. The principle is similar to the convolution operation in convolutional neural networks.

The GCN is viewed as a kernel (GCN as kernel (GCN-AK)), and a new node representation vector is obtained by convolving it with the initial feature vector of the nodes in the subgraph. Specifically, the GCN is used as a subgraph encoder. Then, GCN-AK computes $h_G$ by Eq. (4)

4 $$\begin{aligned} &h_u^{l+1}={GCN}^l{(G}^l\left( N_{rw}\left( u\right) \right) ),&l=0,\cdots ,L-1;h_G=pooling({h_v^l|u\in V}), \end{aligned}$$

where *G* is graph, $G=(V,E)$. $G(N_{rw}(u))$ is the subgraph generated by random walking from the root node *u*. $G^l\left( N_{rw}\left( u\right) \right)$ is the subgraph with hidden features at the *l*-th layer. $h_u^l$ denotes the hidden representation of node *u* in GNN-AK layer *l*. $u\in N_k(u), h_u^{(0)}=\varvec{N}_{init}^u$.

We use ${Subgraph}^l\left( u\right)$ instead of $G^{(l)}(N_k(u))$ to denote the induced subgraph of *u* and use GCN to encode node *i* in subgraph *j* yields the representation vector $Emb(i|{Subgraph}^l\left( j\right) )$. We consider the embedding of all $j\in V$ and all nodes of $i\in {Subgraph}^l\left( j\right)$. That the base GCN can have multiple convolutional layers, and *Emb* refers to the node embeddings at the last layer before global pooling ${pooling}_{GCN}$ that generates subgraph-level encoding.

5 $$\begin{aligned} \begin{aligned} h_u^{(l+1)|subgraph}={GCN}^{(l)}\left( Subgraph^{(l)}\left( u\right) \right) :={pooling}_{{GCN}^{(l)}}\left( \{Emb\left( i|{Subgraph}^{(l)}\left( u\right) \right) |i\in N_k(u)\}\right) , \end{aligned} \end{aligned}$$

We refer to the encoding of the rooted subgraph ${Subgraph}^{(l)}\left( u\right)$ in Eq. (5) as the subgraph encoding. Typical choices of ${pooling}_{{GCN}^{(l)}}$ are *SUM* and *MEAN*. As each rooted subgraph has a root node, ${pooling}_{{GCN}^{(l)}}$ can be additionally realized to differentiate the root node by self-concatenating its own representation, which is “centroid encoding”, resulting in the following realization as each layer of GCN-AK:

6 $$\begin{aligned} h_u^{(l+1)}=FUSE(h_u^{(l+1)|centroid},h_u^{(l+1)|subgraph}) \end{aligned}$$

where $h_u^{(l+1)|centroid} := Emb(u|{(Subgraph}^{(l+1)}\left( u\right)$^40^. *FUSE* is concatenation.

To improve the scalability of the model, we use the subgraph drop strategy, more details refer to. The final node representation vector is $N^u$.

7 $$\begin{aligned} \varvec{N^u}=\sigma (W\cdot h_u^{(l)}+b) \end{aligned}$$

where $\varvec{N}^u\in {\mathbb {R}}^{\psi \times 1}$, and *W* and *b* are learnable parameters. $\psi$ is the final output dimension of the model.

### Output

The node representation vector $\varvec{N}^u$ is obtained based on the processing in the previous subsection, and our model classifies node *u* as a social bot or human by the softmax layer (see as Eq. (8)).

8 $$\begin{aligned} {{\hat{y}}}_i=SoftMax(W\cdot \varvec{N}^u+b),\ \end{aligned}$$

where *W* and *b* are learnable parameters.

The loss function used in model training is the cross-entropy loss function which is commonly used in classification tasks. The proposed model is named as SEGCN and its pseudo-code is given as Algorithm 1.

**Algorithm 1:**
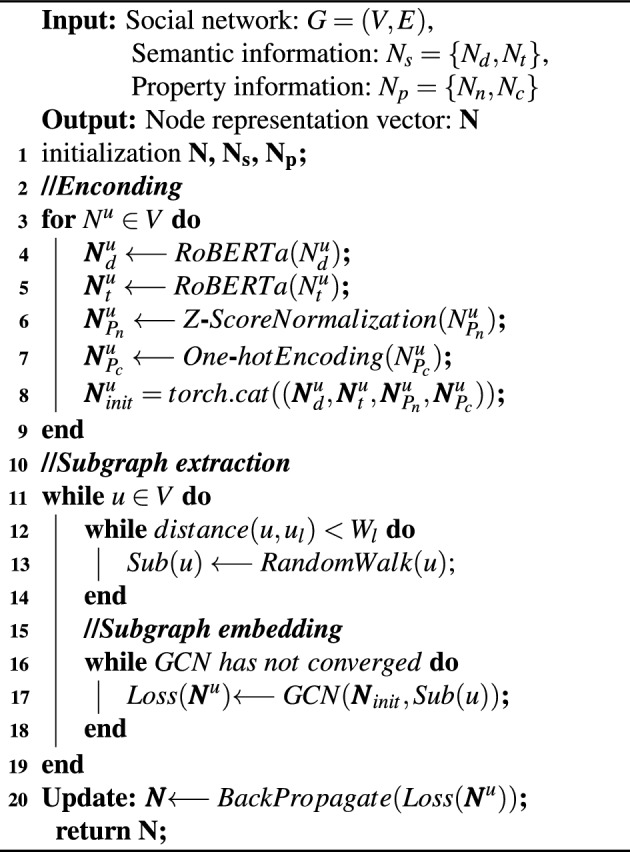
SEGCN

## Experiments

In this section, we perform extensive experiments on two benchmark datasets to validate the performance of the proposed model. All experiments are conducted on a server with Intel (R) Xeon (R) Gold 6234 CPU (4 $\times$ 8 cores, 128 GB, 3.3 GHz) and RTX 3090 (2 $\times$ 24 GB) GPU running Ubuntu 20.04 (64-bit).

### Datasets

The experiments are based on two different publicly available datasets, namely, the TwiBot-20 dataset^53^ and the TwiBot-22 dataset^54^. The TwiBot-20 dataset is a social bot dataset made public by Feng et al.^53^ in 2020, which includes 229,573 Twitter users, 33,488,192 tweets, 8,723,736 user property items and 455,958 following relationships. The TwiBot-22 dataset is a larger social bot dataset made public by Feng et al.^54^ in 2022, which includes 1,000,000 Twitter users (human: 860,057, bot: 139,943), 86,764,167 tweets and 170,185,937 following relationships. An overview of the datasets is presented in Table 2.

**Table 2:** Overview of the benchmark dataset.

Datasets	Total account	Bot account	Human account
TwiBot-20^53^	229,573	5273	6589
TwiBot-22^54^	1,000,000	139,943	860,057

### Baseline methods

In this section, we give a brief introduction of the baseline bot detection models compared with our model.

#### Deepwalk^55^

Deepwalk is a graph embedding algorithm that combines random walk and word2vec, which is able to represent the nodes in a graph as a vector containing potential information. It is widely used in downstream tasks such as node classification, link prediction, and community discovery.

#### Node2vec^56^

Node2vec is a graph embedding model that integrates node structure equivalence and neighbor similarity. Specifically, it introduces breadth-first search (BFS) and depth-first search (DFS) to capture the homogeneity and structural equivalence of nodes, and can be seen as the Deepwalk model that combines BFS and DFS random walks.

#### GCN^44^

GCN is a kind of MPNN. MPNN aggregates the information of neighboring nodes to update the information of central nodes, and it extends the convolution operator to the field of irregular data to realize the connection between the graph and the neural network. It has been widely used for tasks such as node classification, and link prediction.

#### GAT^57^

GAT follows the same message-passing paradigm, which introduces an attention mechanism that takes into account the differences in the influence of neighboring nodes on the central node. It is also widely used for downstream tasks such as link prediction, node classification and graph clustering.

#### Bot2vec^34^

Bot2vec is a social bot detection algorithm using only structural features proposed by Pham et al. in 2021. It is an improved version of Node2vec that introduces community detection algorithms to capture the structural equivalence of nodes.

#### SATAR^32^

SATAR is a self-supervised Twitter account representation model combining account semantic information, property information and neighbor information proposed by Feng et al.^32^. It achieved very good results in the task of detecting novel bots.

#### BotRGCN^11^

BotRGCN is an RGCN-based social bot detection model and it is similar to GCN following the message passing paradigm. Compared to GCN which aggregates on undirected graphs, it can aggregate information about surrounding neighbors in a directed graph format.

#### RFGNN^48^

RFGNN is a method that combines Random Forest and GNNs, which employs GNNs as the base classifiers to construct a random forest, effectively combining the advantages of ensemble learning and GNNs to improve the accuracy and robustness of the model. We use the best-performing RF-RGCN model in RF-GNN as our comparison method. Notably, this method utilizes the BERT model to extract semantic features from tweets and account descriptions.

#### RFGNN-R

RFGNN-R, in comparison to RFGNN, uses the RoBERTa model to extract semantic features, meaning that its method of feature extraction beyond structural features remains consistent with that of GCN, GAT, SATAR, BotRGCN, and our model.

To explicitly compare with the detection models, we present an overview of the account features used by each model in Table 3. Deepwak/Node2vc/Bot2vec exploit the structural features of accounts, and GCN, GAT, SATAR, BotRGCN and our model all exploit the semantic features, property features, and structural features of accounts. “–” is None.

**Table 3:** Overview of account information used by the compared models.

Model	Published	Semantic	Property	Structure
Deepwalk	2014	–	–	$\checkmark$
Node2vec	2017	–	–	$\checkmark$
Bot2vec	2022	–	–	$\checkmark$
GCN	2017	$\checkmark$	$\checkmark$	$\checkmark$
GAT	2018	$\checkmark$	$\checkmark$	$\checkmark$
SATAR	2021	$\checkmark$	$\checkmark$	$\checkmark$
BotRGCN	2021	$\checkmark$	$\checkmark$	$\checkmark$
RFGNN	2023	$\checkmark$	$\checkmark$	$\checkmark$
Ours	–	$\checkmark$	$\checkmark$	$\checkmark$

**Table 4:** Overview of models’ parameter configuration.

Parameter	Deepwalk	Node2vec	Bot2vec	GCN	GAT	SATAR	BotRGCN	RFGNN	Ours
Network layers	–	–	–	2	2	1	2	2	2
Dropout value	–	–	–	0.3	0.3	0.6	0.3	0.3	0.3
Embedding size	128	128	128	128	128	128	128	128	128
Learning rate	–	–	–	0.001	0.001	0.01	0.001	0.001	0.01
Weight decay	–	–	–	0.005	0.005	0	0.005	0.005	0.005
Optimizer	–	–	–	AdamW	AdamW	SGD	AdamW	AdamW	AdamW
Epochs	–	–	–	100	100	100	100	100	100
Window size	7	7	7	–	–	–	–	–	–
Negative sampling	5	5	5	–	–	–	–	–	–
Walk length	30	30	30	–	–	–	–	–	30
Number of walks	20	20	20	–	–	–	—	–	–
Return parameter	–	1	1	–	–	–	–	–	–
In-out parameter	–	1	1	–	–	–	–	–	–

### Implementation details

We conducted the experiments based on the source code provided by the authors. For model-specific parameters, we used the default configuration of the code, and we tried our best to ensure that the common parameters have the same configuration. The parameter configuration of all models in the experiments is shown in Table 4. “–” is None. The source code for these baseline models can be found in the original paper as well as in TwiBot-22.

### Experimental results

To validate the performance of the models, we followed the data setting approach used in baseline models such as BotRGCN, SATAR and Bot2vec. The Deepwalk/Node2vec/Bot2vec models in both public datasets are trained with 90% of the data and tested with the remaining 10% . Both GCN/GAT/SATAR/BotRGCN/RFGNN and our model use 70% of the data as the training set, 20% of the data as the validation set, and the remaining 10% of the data as the testing set. The training of neural network is stochastic to some extent, so the learned model weights and errors can vary slightly after each iteration even with the fixed hyperparameters and data splits. In order to avoid the randomness in the training process, the models are trained and tested for 5 iterations but with the same partitioned data. The average performance over the repeated experiments is reported as the final result, which smooths out the random fluctuations and provides a more stable assessment of model effectiveness. Accuracy, F1-Score and Precision are used as evaluation metrics and experiments are conducted on three benchmark datasets. The experimental results are shown in Table 5, where the best results are in bold.

**Table 5:** Performance comparison of multiple social bot detection models on three benchmark datasets (%).

Model	Twitbot-20				TwiBot-22			
	Accuracy	F1-Score	Precision	AUC	Accuracy	F1-Score	Precision	AUC
Deepwalk	56.31 ± 1.34	61.13 ± 0.87	53.27 ± 1.26	57.71 ± 0.73	51.87 ± 1.65	37.94 ± 1.41	49.17 ± 0.76	50.28 ± 0.91
Node2vec	60.66 ± 1.03	66.05 ± 1.17	59.23 ± 0.89	61.81 ± 0.87	57.11 ± 1.25	39.27 ± 1.31	55.97 ± 0.97	55.78 ± 1.07
Bot2vec	63.28 ± 0.87	71.47 ± 1.04	63.18 ± 0.71	60.37 ± 1.37	59.14 ± 0.83	41.08 ± 1.16	57.26 ± 0.73	49.81 ± 2.37
GCN	74.64 ± 0.24	77.03 ± 0.37	73.17 ± 0.17	83.07 ± 0.48	72.39 ± 0.51	44.80 ± 0.35	71.19 ± 0.27	72.78 ± 0.42
GAT	83.27 ± 0.32	85.25 ± 0.44	81.26 ± 0.29	84.63 ± 0.56	78.36 ± 0.41	55.86 ± 0.39	72.23 ± 0.25	73.47 ± 0.33
SATAR	84.02 ± 0.17	86.07 ± 0.24	81.50 ± 0.18	90.88 ± 0.27	78.71 ± 0.42	57.10 ± 0.26	74.07 ± 0.13	79.26 ± 0.67
BotRGCN	84.61 ± 0.38	87.07 ± 0.43	83.79 ± 0.24	91.46 ± 0.26	79.66 ± 0.14	57.50 ± 0.12	74.81 ± 0.21	78.21 ± 0.56
RFGNN	83.92 ± 0.21	83.37 ± 0.27	82.19 ± 0.44	88.67 ± 0.48	78.61 ± 0.32	55.67 ± 0.41	72.86 ± 0.56	76.87 ± 0.51
RFGNN-R	85.03 ± 0.69	87.96 ± 0.57	84.11 ± 0.51	91.67 ± 0.63	80.37 ± 0.46	57.97 ± 0.58	75.33 ± 0.51	79.61 ± 0.63
Ours	87.01 ± 0.08	88.74 ± 0.13	85.83 ± 0.06	93.79 ± 0.32	82.71 ± 0.16	59.31 ± 0.12	77.23 ± 0.17	82.31 ± 0.49

**Figure 3:**
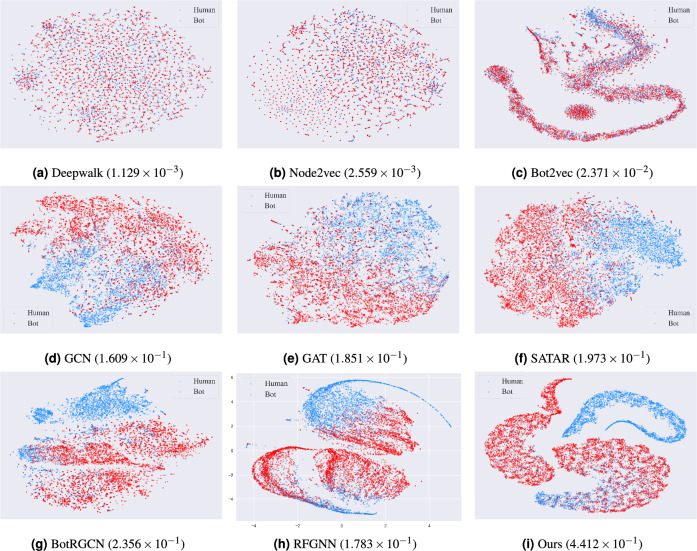
Visualization of human-bot user representations of the TwiBot-20 dataset by various models via t-SNE 2D projections and the corresponding homogeneity score.

**Figure 4:**
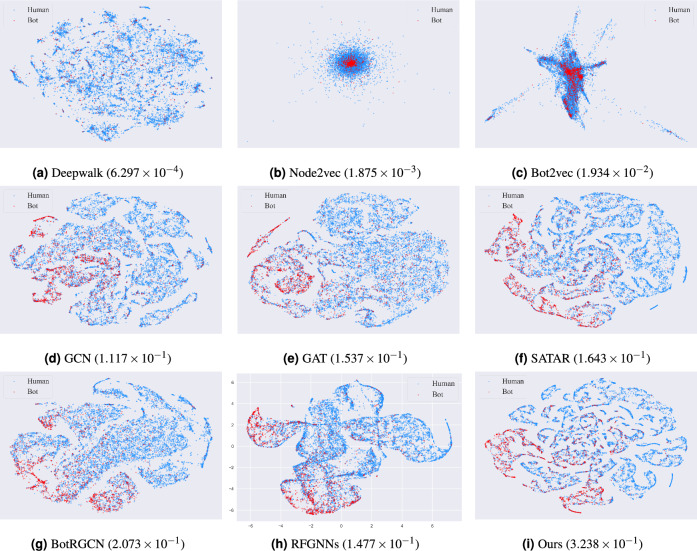
Visualization of human-bot user representations of the TwiBot-22 dataset by various models via t-SNE 2D projections and the corresponding homogeneity score.

As seen in Table 5, the Accuracy of the social bot detection model (Deepwalk/Node2vec/Bot2vec) using only graph structure features is below 0.65 on the Twibot-20 dataset, which may be ascribed to the following reasons. Only 20 neighbor nodes (10 Following and 10 Followers) were extracted for each account in the Twibot-20 dataset, and the structural features of the accounts were impaired. Such models use only structural features which allow novel bots to evade detection. The social bot detection models that simultaneously utilize account property features, semantic features, and structural features all have an accuracy of over 74% on the Twibot-20 dataset, which improves the detection accuracy by more than 10% than purely utilize graph structural features, indicating the desirability of combining multiple types of features for social bot detection. Compared with the GCN model, GAT and SATAR introduced attention mechanisms, and the effect was improved by more than 8.6%. BotRGCN divides the edge into Following edge and Follower edge, aggregates the surrounding neighbor information according to different relationships, and the accuracy is improved by about 9.9%; Our model uses subgraph encoding to improve accuracy by about 12.4%. These phenomena indicate that changing the node aggregation method affects the performance of the model. Compared with the BotRGCN, our model’s detection accuracy improves by about 2.4%, compared with the RFGNN-R, our model’s detection accuracy improves by about 2.0%, indicating that the design idea of the subgraph encoding-based graph convolutional network social bot detection model is feasible.

To justify why the proposed model has better performance, we use the t-SNE 2D visualization technique to visualize the embedding vectors and the corresponding homogeneity score obtained by each model on the TwiBot-20 dataset and TwiBot-22, as illustrated in Figs. 3 and 4. The t-SNE visualization results can reflect the quality of model training to a certain extent^11,12,32,34^. A higher homogeneity score means the samples are better clustered. It can be observed from Figs. 3 and 4 that our model achieves the highest homogeneity score and the embedding vector obtained from our model training is more beneficial for the social bot detection task.

**Figure 5:**
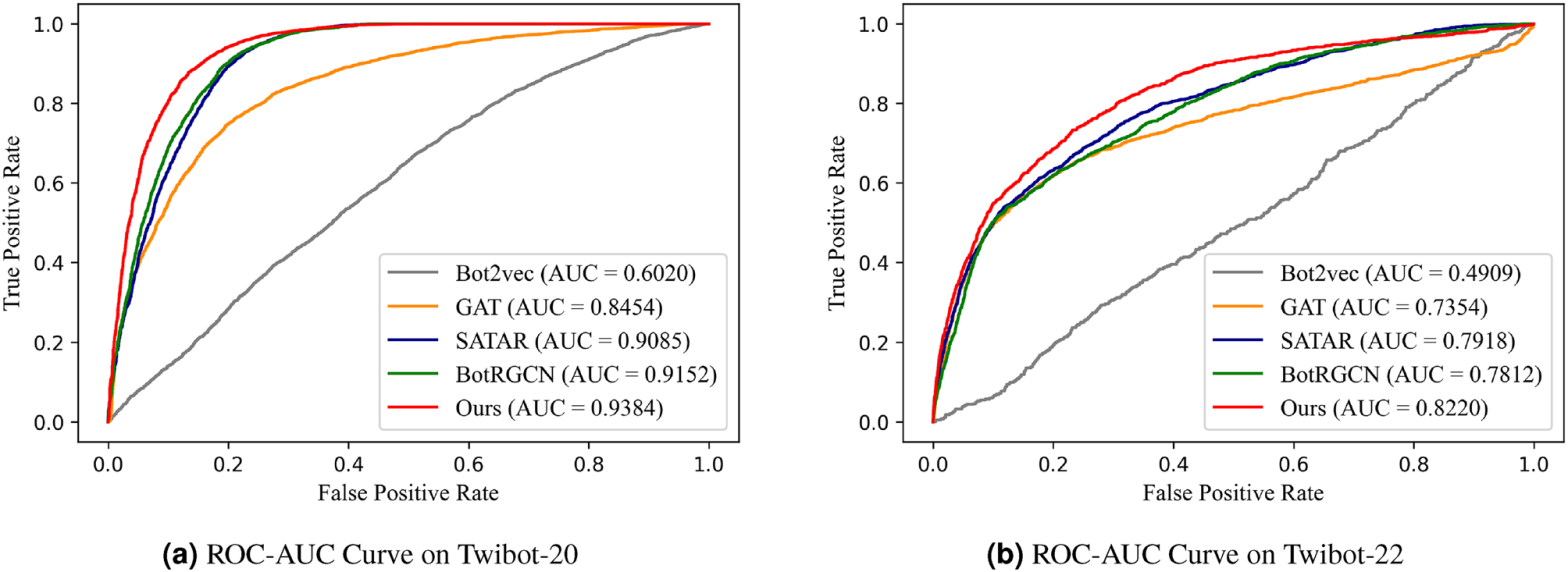
The ROC-AUC curve on two benchmark datasets.

**Figure 6:**
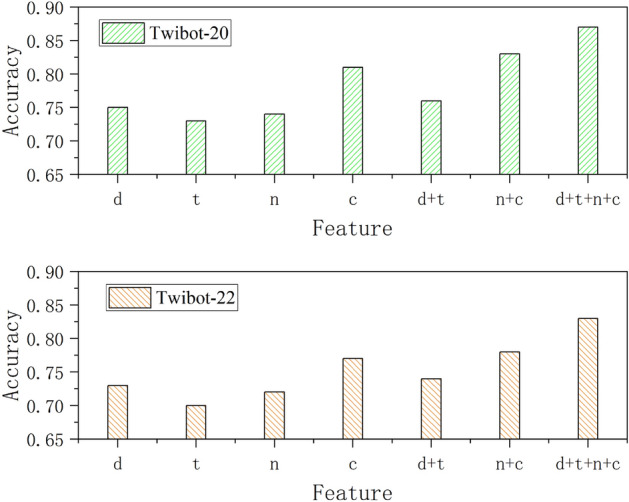
Illustration of accuracy when using various combination of the features for the training of the SEGCN model. The features used are accounts’ description features (d), tweet feature (t), numerical features (n) and category features (c).

In addition, we selected five representative models and plotted the ROC-AUC curves of each model on the Twibot-20 and Twibot-22 datasets based on the SVM classifier (Fig. 5). Observing the ROC-AUC curves, the one corresponding the node representation vectors learned by our model has the largest area under the curve, which indicates the proposed model has a stronger expressive power than the compared models.

#### Ablation experiment on features

To investigate the effect of different types of features on the detection performance of our model, we conducted feature ablation experiments on two datasets. After adding account description features (d), tweet semantic features (t), numeric features (n), and category features (c) to SEGCN, the detection accuracy of the model are shown in Fig. 6. By comparing “d”, “t”, “n” and “c”, we can see that the category features have a greater impact on the model performance, which may be due to the fact that both datasets have more important category features such as whether they are authenticated or not. The accounts that are authenticated are usually human accounts. Most importantly, the best detection performance is achieved by “d+t+n+c”, which validates that all of the four types of features are necessary for social bot detection.

In general, the use of subgraph encoding can capture the differences of structural features in subgraphs and improve the expressive power of GCN, and a large number of experiments showed the good performance of SEGCN. It should be noted that the proposed model is a general social bot detection framework, which is applicable for furthermore meaningful features. It can also adjust the features dynamically according to the development of social bot, which has great potential for industrial applications.

## Discussion

This section discusses the differences between our research and the existing ones. The investigation in^10^ and extensive experimental results in “Experiments” shows that the evolution of social bots made social bot detection methods using only a single type of feature less effective in detecting novel bots. The existing social bot detection methods using multiple types of features have yielded promising results in detecting novel bot tasks, but they ignore the fact that the MPNN’s expressive power is upper-bounded by the 1-WL isomorphism test^15^. The experimental results in Table 5 shows that compared with classical GCN, the subgraph coding can better capture the structural features of nodes in the social bot detection task, indicating that the subgraph encoding can improve the expression ability of GCN.

The most significant difference between our model and the existing ones is that subgraph coding method is introduced to improve the performance of social bot detection. To explicitly compare with the detection models, we present an overview of the account features used by each model in Table 3. The Deepwalk^55^, Node2vec^56^ and Bot2vec^34^ utilize the structure features of the account. GCN^44^, GAT^57^, SATAR^32^, BotRGCN^11^, RFGNN^48^ and our model all exploit the semantic features, property features, and structural features of the account. However, our model uses subgraph encoding to improve the expressiveness of the GCN.

## Conclusion

In this paper, we propose a subgraph encoding based graph convolutional network model for social bot detection, named SEGCN, which uses subgraph encoding to improve the expressive power of graph convolutional networks and uses multiple types of features simultaneously for social bot detection. To the best of our knowledge, this is the first work using subgraph encoding based graph convolutional networks for social bot detection. Experimental results on two benchmark datasets show that the model achieves better performance than the SOTA approach and effectively improves the expressive power of GCN. However, the application of the proposed method in the real world social platform, for instance, Twitter (now called X ), is facing more difficulty, because some of the data that needed to evaluate the social account is not free to access anymore. Nevertheless, our method provides a generalized framework for social bot detection, and social platforms and individuals can refer to this pipeline to detect the social bots. In the future, we will try to investigate the construction of heterogeneous graphs to detect social bots using accounts in social networks with multiple types of activity relationships.

## Data Availability

The Twibot-20 dataset and the Twibot-22 dataset are used to support the findings of this study, which are available at “https://github.com/BunsenFeng/TwiBot-20” and “https://github.com/LuoUndergradXJTU/TwiBot-22”, respectively.
